# *Aloe vera* Gel: Effective Therapeutic Agent against Multidrug-Resistant *Pseudomonas aeruginosa* Isolates Recovered from Burn Wound Infections

**DOI:** 10.1155/2015/639806

**Published:** 2015-07-22

**Authors:** Mehdi Goudarzi, Maryam Fazeli, Mehdi Azad, Sima Sadat Seyedjavadi, Reza Mousavi

**Affiliations:** ^1^Department of Microbiology, School of Medicine, Shahid Beheshti University of Medical Science, Tehran 1985717443, Iran; ^2^WHO Collaborating Center for Reference and Research on Rabies, Pasteur Institute of Iran, Tehran 1316943551, Iran; ^3^Department of Medical Laboratory Sciences, School of Paramedicine, Qazvin University of Medical Sciences, Qazvin 1651135779, Iran; ^4^Department of Medical Mycology, Pasteur Institute of Iran, Tehran 1316943551, Iran; ^5^Department of Medical Laboratory Sciences, School of Paramedicine, Shahid Beheshti University of Medical Sciences, Tehran 1985717443, Iran

## Abstract

*Objective*. *Aloe vera* is an herbal medicinal plant with biological activities, such as antimicrobial, anticancer, anti-inflammatory, and antidiabetic ones, and immunomodulatory properties. The purpose of this study was investigation of *in vitro* antimicrobial activity of *A. vera* gel against multidrug-resistant (MDR) *Pseudomonas aeruginosa* isolated from patients with burn wound infections. *Methods*. During a 6-month study, 140 clinical isolates of *P. aeruginosa* were collected from patients admitted to the burn wards of a hospital in Tehran, Iran. Antimicrobial susceptibility test was carried out against the pathogens using the *A. vera* gel and antibiotics (imipenem, gentamicin, and ciprofloxacin). *Results*. The antibiogram revealed that 47 (33.6%) of all isolates were MDR *P. aeruginosa*. The extract isolated from *A. vera* has antibacterial activity against all of isolates. Also, 42 (89.4%) isolates were inhibited by *A. vera* gel extract at minimum inhibitory concentration (MIC) ≤ 200 *µ*g/mL. MIC value of *A. vera* gel for other isolates (10.6%) was 800 *µ*g/mL. All of MDR * P. aeruginosa* strains were inhibited by *A. vera* at similar MIC_50_ and MIC_90_ 200 *µ*g/mL. *Conclusion*. Based on our results, *A. vera* gel at various concentrations can be used as an effective antibacterial agent in order to prevent wound infection caused by *P. aeruginosa*.

## 1. Introduction

*Pseudomonas aeruginosa* (*P. aeruginosa*) is increasingly recognized as one of the important nosocomial pathogens leading to severe infections especially in hospitalized patients in burn wards [1]. This opportunistic and highly resistant pathogen is responsible for a variety of nosocomial infections, including urinary or wound infections, bacteremia, endocarditis, and in some conditions death.* P. aeruginosa* infections in immunocompromised, debilitated patients, cystic fibrosis, and hospitalized burn patients are associated with increased rates of mortality and morbidity [1, 2]. The unselective and extensive use of antibiotics is highly considered as the major cause of invasive procedures. Accordingly, development of resistance mechanisms either intrinsic or acquired has promoted the rapid development of multiple resistances among* P. aeruginosa* isolates in the clinical settings [2]. The rapid increase of drug resistance in clinical isolates of* P. aeruginosa* is a growing concern among hospitalized patients [3, 4]. During the past several decades, several different epidemiological studies indicated that multiple resistances among* P. aeruginosa* clinical isolates are increasing [5]. The widespread multidrug-resistant (MDR)* P. aeruginosa* strains not only lead to increased economic burden, but also can directly threaten the life of the patient. Ciprofloxacin, gentamycin, and imipenem are routinely used for treatment of the* P. aeruginosa* burn wound infections in Iran. Recently, overusing of imipenem in comparison with fluoroquinolones, beta lactams, and aminoglycoside, due to their resistance, demonstrated a risk of resistant to this antibiotic in* P. aeruginosa* isolated from burned patients [1]. Nowadays, multiple resistance mechanisms of* P. aeruginosa* isolates present serious therapeutic challenges for treatment [6]. Although antibiotics are routinely used for prevention of the* P. aeruginosa* burn wound infections, due to widespread MDR among* P. aeruginosa* strains leading to therapeutic failures, they are not recommended for regular prevention of burn wound infections associated with the bacteria [1–3]. The use of newer antibiotics, moist exposure therapy, mafenide acetate, antimicrobial peptides, and acticoat AB dressing (high-density polyethylene mesh coated with nanocrystalline silver) could be used for treatment of wound infected with MDR* P. aeruginosa* [1]. Generally, therapeutic strategies should be revised to control* P. aeruginosa* infections with emphasis on the use of extract and biologically active compounds isolated from the herbal plants. During the past several decades, many studies both* in vivo* and* in vitro* have demonstrated antibacterial activities of medicinal plants. According to estimates by World Health Organization (WHO), in developing countries, about 80% of the population mainly relies on traditional therapies and use of plant extracts as the main medicinal source to treat various infectious diseases [7, 8]. In the recent years, extracts or oils of medicinal plants with antimicrobial and anti-inflammatory effects have been used for treatment of many human infectious diseases.* Aloe vera* (*A. vera*) is one of these well-known medicinal plants [9].* A. vera* is a cactus-like perennial, drought resistant, succulent plant belonging to the Liliaceae family, of which there are over 360 known species. The elongated and pointed leaves of plant contain two distinct products: yellow latex (exudate) and clear mucilaginous gel (*A. vera* gel).* A. vera* gel is revealed after removal of the thick outer cuticle [10]. The gel consists of 99.3% water and the remaining 0.7% containing a range of active compounds including polysaccharides, vitamins, amino acids, phenolic compounds, and organic acids [11]. Overall, more than 75 active ingredients have been identified from the inner gel [12].* A. vera* was first used in the 1930s to heal radiation burns [13]. To date, there are many reports of its beneficial properties in human, so that it is used for pharmaceutical, food, and cosmetic industries.* A. vera* gel has been used in gastrointestinal disorders, sunburn, and wounds since ancient times. Furthermore, various* in vitro* and* in vivo* studies on* A. vera* have demonstrated that it possesses several biological activities, such as anti-inflammatory, antioxidant, immune modulating, and cell growth stimulatory activity as well as antibacterial, antiviral, and antifungal properties [14].

Although antimicrobial properties of* A. vera* gel are primarily recognized, there is still little information on the uses of it and it needs to be more evaluated. The purpose of this study was to investigate the* in vitro* antimicrobial activity of* A. vera* gel against MDR* P. aeruginosa* isolated from burn wound infections.

## 2. Materials and Methods

### 2.1. Bacterial Strains

The present descriptive study was performed on burn cases that were hospitalized in a hospital in Tehran, Iran. A total of 140 clinical isolates of* P. aeruginosa* were collected from November 2014 to April 2015. Nonduplicate isolates were obtained from the burn wound specimens. The identification of isolates was carried out based on standard microbiological procedures such as Gram stain, catalase test, oxidase test, ornithine decarboxylase, arginine dihydrolase, growth at 42°C, growth on Cetrimide agar medium (Liofilchem, Italy), O/F (Oxidation-Fermentation) test, and pigment production [16]. The confirmed strains isolated as* P. aeruginosa* were then stored at −70°C in Tryptic Soy Broth (TSB) (Merck, Germany) containing 20% (v/v) glycerol for further investigation.

### 2.2. Preparation of* A. vera* Gel

The plant of* A. vera* (leaves) was harvested from a local farm in the south Iran in January 2015. The plant was identified in the Research Center of Medicinal Plants. The collected mature and fresh leaves of* A. vera* were thoroughly washed with sterile distilled water to remove dirt and their thick epidermidis was then dissected longitudinally into pieces. The colourless parenchymatous tissue (aloe gel) was collected in a sterile container. One hundred grams of the gel was mixed in one liter of 2% dimethyl sulfoxide (DMSO) and kept at 4°C, being used as a stock solution for Minimum Inhibitory Concentration (MIC) assay.

### 2.3. Minimum Inhibitory Concentration (MIC) Assay

#### 2.3.1. MIC of Synthetic Antibiotics

The antimicrobial susceptibility test of isolates was determined by estimating MIC of the 3 antibiotics using microbroth dilution method according to the Clinical Laboratory and Standards Institute (CLSI, 2012) guidelines [15]. The MIC was defined as the lowest concentration of an antimicrobial agent that will inhibit the visible growth of the tested isolate. MIC_50_ was defined as the MIC required to inhibit the growth of 50% of organisms and MIC_90_ was defined as the MIC required to inhibit the growth of 90% of organisms. The following antimicrobial agents were used in this study: imipenem, gentamicin, and ciprofloxacin (Sigma-Aldrich, St. Louis, Mo). The concentrations range of the used antimicrobial agents was, respectively, imipenem from 0.5 to 32 *μ*g/mL; gentamicin from 1 to 64, and ciprofloxacin from 0.5 to 16 *μ*g/mL. The breakpoints for different antibiotics were as follows: imipenem 8 *μ*g/mL, gentamicin 16 *μ*g/mL, and ciprofloxacin 4 *μ*g/mL.* P. aeruginosa* ATCC 27853 was used as the control strain for antimicrobial susceptibility test.

#### 2.3.2. MIC of* Aloe vera* Gel

In order to determine the MIC of* A. vera* gel against* P. aeruginosa* isolated from burned patients, broth microdilution method was carried out in 96-well cell culture plates. In brief, the bacterial suspensions of the clinical isolates were obtained from overnight cultures. The turbidity of each bacterial suspension was adjusted equivalent to a number 0.5 McFarland standard (approximately 1.0 × 10^8^ CFU/mL). This suspension was further diluted 1 : 100 (10^6^ CFU/mL) in the broth media. Twofold dilutions of the* A. vera* gel stock solution were prepared in the appropriate broth culture media. Aliquots (200 *μ*L) of each dilution (25, 50, 100, 200, 400, and 800 *μ*g/mL) were dispensed in 96-well cell culture plates. One hundred microliters of each bacterial suspension was added to each well and incubated at 37°C overnight. Wells with DMSO (sterility control) and bacterial suspension (drug-free control) were also used as controls [17]. To estimate MIC of* A. vera* gel, the absorbance of each well was measured at 595 nm. MIC level was defined as the lowest concentration at which no growth was observed.

### 2.4. Statistical Analysis

Statistical analysis was performed using SPSS software for Windows, version 17.0 (SPSS Inc., Chicago, IL).

## 3. Results

### 3.1. Antibacterial Activity of Antimicrobial Agents

During the 6-month study period, 140* P. aeruginosa* isolates were recovered from hospitalized burns patients. Of the 140 isolates included in the study, 98 isolates (70%) had been isolated from males and 42 isolates (30%) had been isolated from females. The median age was 46.4 years with an age range from 5-month to 69-year old. The result of the antimicrobial susceptibility testing for 140* P. aeruginosa* clinical isolates using microdilution method for three antibiotics tested revealed that resistance to ciprofloxacin was in 102 (72.8%), to imipenem was in 66 (47.1%), and to gentamicin was in 74 (52.8%) isolates. Resistance rates varied among* P. aeruginosa* clinical isolates from 47.1% for imipenem to 72.8% for ciprofloxacin. MDR was defined as resistance to 2 or more antibiotics [4]. Forty-seven isolates (33.6%) were characterized as the MDR* P. aeruginosa* strains.

### 3.2. Antibacterial Activity of* Aloe vera* Gel

The results obtained about antibacterial activities of* A. vera* gel showed that all of MDR* P. aeruginosa* strains except five of them were inhibited by* A. vera* gel extract at MIC ≤ 400 *μ*g/mL. The MIC values of A.* vera* gel for the remaining five (10.6%) isolates were 800 *μ*g/mL. Undiluted* A. vera* gel exhibited inhibitory effect on all of MDR isolates. None of the MDR isolates were sensitive to dilutions of* A. vera* gel less than 25 *μ*g/mL.* In vitro* susceptibility of the MDR* P. aeruginosa* strains to three antibiotics was performed and* A. vera* gel with the range of MIC_50_ and MIC_90_ are summarized in Table 1.

## 4. Discussion

In the our study, all of MDR* P. aeruginosa* strains (except five of them) were found to be sensitive to* A. vera* gel extract at MIC ≤ 400 *μ*g/mL. Also, the data obtained in this study showed a good antibacterial activity of* A. vera* gel extract in different concentration against MDR* P. aeruginosa* isolated from burned patients despite many published reports in this regard. These controversial results could be attributed to difference in susceptibility testing method, difference in extraction process, stresses heat imposed on the gel, and the number of pharmacologically active compounds including anthraquinones, anthracene, anthranol, aloin, aloe mannan, aloetic acid, aloe emodin, aloeride, chrysophanic acid, resistanol, and saponin [18, 19].

Many medicinal plants as potential source of novel antimicrobial compounds,* A. vera* being the most important one, are widely used by large proportion of the Iranian population for treating burn wound infections [20–22]. These results regarding antibacterial activity are in conformity with earlier findings of Antonisamy et al. who found varied antibacterial antifungal activity of DMSO gel extracts of* A. vera* against five bacterial cultures of* Bacillus subtilis, Salmonella typhi, Escherichia coli,* and* Staphylococcus aureus* and three fungal cultures of* Aspergillus fumigatus, Candida albicans,* and* Penicillium sps*. The results showed that DMSO gel extracts of* A. vera* had highest degree of activity against the selected pathogens and the degree of inhibition varied depending upon the concentration of the extract [23–26]. Scientific evidences have proven that the constituents of gel and leaf are distinct from each other. Some of investigators believe that the gel is more active than the leaf, but one clear feature is that the gel and leaf can complement one another in their medicinal capabilities [27]. Surprisingly, there are contradictory studies about inhibitory effect of gel, leaf, and juice of* A. vera*. In 2010, Anderl et al. showed that both gel and leaf inhibited the growth of* S. aureus* isolated from wound infection while the gel has no effect on* P. aeruginosa* isolates [28].

Several studies exhibited higher level of antibacterial activity of* A. vera* gel against fungi strains [29]. Agarry et al. conducted a study to compare the antimicrobial activities of the gel and leaf of* A. vera* against* P. aeruginosa*,* S. aureus*,* Microsporum canis*,* Trichophyton schoenleinii*,* T. mentagrophytes,* and* C. albicans*. The results presented the significant antibacterial activity of both the gel and the leaf against* S. aureus*. The leaf possesses inhibitory effects on both* P. aeruginosa* and* C. albicans* while only the gel inhibited the growth of* T. mentagrophytes* [27]. Investigation of antimicrobial activities and MIC of* A. vera* gel and* Aloe vera* juice in the study of Kaithwas et al. in 2008 clearly demonstrated that* A. vera* juice has higher and broader spectrum of antimicrobial activity than* A. vera* gel [30]. This difference in activity of the juice as compared to gel could be attributed to presence of greater amount of the anthraquinones and phenolic antioxidants in the* A. vera* juice.

There are differences in the antimicrobial effects of various extracts of* A. vera*. Irshad et al. showed the* A. vera* extract of Methanol had the maximum antibacterial activity as compared to other solvents, ethanol, and distilled water [31]. The study of Ibrahim et al. in 2011, in order to investigate phytochemical analysis and antimicrobial evaluation of* A. vera* gel against some human and plant pathogens by disc diffusion method, was done with three different forms (ethanol, acetone, and aqueous extracts). The antibacterial and antifungal activity of the acetone extract was found to be more effective than ethanol and aqueous extracts [32]. Pandey and Mishra reported that there was no inhibitory effect of aqueous extract on the Gram negative bacteria and weak inhibitory effect on Gram positive bacteria [33]. Differences in the antimicrobial effects of various extracts of* A. vera* may be attributed to different solubility of various compounds found in* A. vera*, particularly, some solvents that have a specific antifungal or antimicrobial activity.

Although known ingredients in the* A. vera* leaf gel are obviously present in small mounts (about 1%), they can be linked to work together and create synergistic effect so that the total effect is greater than what would be expected from the individual effect of each substance [34–36].

## 5. Conclusion

Our study supports the view that* A. vera* gel could be active against* P. aeruginosa* in wound infections at various concentrations and the use of it at optimum concentrations can help better therapy of many microbial diseases. Further investigations are required to identify bioactive components of* A. vera* gel and its effect on wide range of bacteria and fungus including the pathogenic strains. It is hoped that this study would lead to the development of aloe gel usage as a main medicinal source to treat various infectious diseases.

## Figures and Tables

**Table 1:** Summary of the antibacterial activities of *A*. *vera* gel and antimicrobial agents against clinical isolates of *P*. *aeruginosa*.

Agent	MIC (*μ*g/mL)			Number (%) of isolates			MIC interpretive breakpoints^a^		
	Range	50%	90%	S	I	R	S	I	R
*Aloe vera* gel	25–800	200	200	—	—	—			
Ciprofloxacin	0.5–16	4	8	33 (23.6)	5 (3.6)	102 (72.8)	≤1	2	≥4
Gentamicin	1–64	8	32	61 (43.6)	5 (3.6)	74 (52.8)	≤4	8	≥16
Imipenem	0.5–32	4	16	70 (50)	4 (2.9)	66 (47.1)	≤2	4	≥8

^a^MIC breakpoints applied were those recommended for *P*. *aeruginosa* by the Clinical and Laboratory Standards Institute (CLSI) [15].
